# Prevalence and Risk Factors of Human Papillomavirus in Male Patients: A Systematic Review and Meta-Analysis

**DOI:** 10.3390/ijerph15102210

**Published:** 2018-10-10

**Authors:** María Inmaculada Rodríguez-Álvarez, Jose L. Gómez-Urquiza, Husein Husein-El Ahmed, Luis Albendín-García, Juan Gómez-Salgado, Guillermo A. Cañadas-De la Fuente

**Affiliations:** 1 Hospital of Baza, Department of Dermatology, Andalusian Health Service, 18016 Granada, Spain; roxhibac@gmail.com (M.I.R.-Á.); huseinelahmed@hotmail.com (H.H.-E.A.); lualbgar1979@ugr.es (L.A.-G.); 2 Department of Nursing, University of Granada, 18016 Granada, Spain; gacf@ugr.es; 3 Department of Nursing, University of Huelva, 21007 Huelva, Spain; jgsalgad@gmail.com; 4 Safety and Health Posgrade Program, Espíritu Santo University, Guayaquil 091650, Ecuador

**Keywords:** epidemiology, risk factors, men, papillomavirus, prevalence, health promotion

## Abstract

Human papillomavirus (HPV) is one of the most prevalent sexually transmitted infections. Although the research focus has been on women, men are also affected. Thus, the aim was to estimate the prevalence of HPV in men and to analyse its risk factors. A systematic review with meta-analysis was performed. The main health science databases were consulted. The search terms were was: “papilloma virus AND (prevalence OR risk factors) AND men”. The final sample of studies was *n* = 16 and the men sample for the meta-analysis was *n* = 18,106. The meta-analysis revealed a prevalence of 49% (95% Confidence Interval (CI): 35–64%) of any type of human papillomavirus in men and 35% (95% CI: 26–45%) of high-risk human papillomavirus in men. The included studies showed that stable sexual habits, circumcision and condom use are protective factors against HPV. In addition, there is a certain positive association with tobacco use and the early initiation of sexual intercourse. In conclusion, the prevalence of HPV in men is high. The risk factors for HPV infection are sexual promiscuity, early sexual debut, absence of circumcision, lack of condom use and smoking. Further study in this field about the effectiveness of the vaccine and health education should be conducted.

## 1. Introduction

Human genital papillomavirus (HPV) is a common sexually transmitted infection (STI) which has become a major source of morbidity and mortality worldwide [1]. More than 100 types of HPV have been identified to date. Although most are harmless, about 30 types are associated with an increased risk of cancer. Those oncogenic HPV are classified as low or high risk subtypes. HPV infections usually clear without intervention, although low-risk HPV can cause genital warts. In women, high-risk HPV can provoke cancer in several locations such as the cervix, vulva, vagina and anus. In men, HPV can lead to cancer of the anus and of the penis [2,3]. Additionally, HPV can also produce cancer in the back of the throat, as well as the base of the tongue and tonsils (oropharyngeal cancer) [4,5,6].

Among the low-risk subtypes, the most common serotypes include HPV-6 and HPV-11, which usually cause benign warts and are occasionally associated with non-invasive lesions. In contrast, serotypes HPV-16 and HPV-18 are considered high-risk subtypes, due to their high carcinogenic potential. HPV-16 appears primarily in invasive tumours and in those lesions with a high degree of malignancy, while HPV-18 is associated with poorly differentiated carcinomas and with increased lymph node involvement [4,7].

Most studies of HPV have analysed diagnosis, treatment and prevention in women. Moreover, strategies in sexual and reproductive health programmes in many countries have focused on epidemiological control in women, but they have tended to overlook the role of men in this infection, despite its high prevalence. In fact, it has been suggested that men may constitute a reservoir for inadvertently transmitting infection to women, due to its asymptomatic nature, thus contributing to the persistence of infection and cancer [8]. A further consideration is that the few published studies in this field have reported divergent results.

Several of the risk factors for HPV in men are associated with deficits in hygiene and prevention [9,10]. In fact, HPV vaccines are available against the serotypes, which protect men from most forms of the disease [11]. Although the HPV vaccine is most effective before the first sexual activity, many sexually active men aged from 18 to 26 may also benefit from HPV vaccination [12].

In view of the above considerations, this study was undertaken to perform a meta-analysis of HPV prevalence in men and to analyse the corresponding risk factors. The question that guided this study was: what is the prevalence of HPV in men and which are its risk factors?

## 2. Method

### 2.1. Search Strategy

A systematic review and meta-analysis of the literature was conducted, following the PRISMA [13] recommendations. The following scientific databases were consulted: PubMed, Scopus, CINAHL, LILACS, Proquest Health and Medical Collection, Dialnet and SciELO. The following search term was used, with Medical Subject Headings: “papillomavirus AND (prevalence OR risk factors) AND men”. The search was conducted in January 2018. 

### 2.2. Inclusion/Exclusion Criteria

The analysis included quantitative studies on HPV in men published in English or Spanish, unrestricted by year. Studies lacking quantitative statistical information were excluded, as were studies with mixed samples without independent data on HPV in men, studies that associated HPV with other diseases, and studies focused on the genetics of the virus.

### 2.3. Variables and Data Collection

A data collection notebook was used. For longitudinal studies with more than one HPV evaluation, the first measure obtained in the study population was recorded. The following variables were collected on the characteristics of the sample: year of publication, country of study, type of publication (article vs. doctoral thesis) and study design (cross-sectional or longitudinal).

### 2.4. Selection of Studies, Critical Reading and Level of Evidence

Once the preliminary results obtained from each database, the title and abstract were read. The full text was then read. After this, a forward and reverse search was carried out within the studies included to locate as many documents as possible. Finally, the studies were read critically before the inclusion. The methodological quality was assessed with the checklist proposed by Ciaponni. The levels of evidence and degree of recommendation used were those proposed by the Oxford Centre for Evidence-based Medicine [14].

### 2.5. Data Analysis

Data analysis was performed with the StatsDirect meta-analysis package (version 3, StatsDirect Ltd., Cambridge, UK). The first step was to perform a sensitivity analysis to ensure that none of the studies included produced significant changes in outcomes when excluded. Publication bias were then assessed using Egger’s linear regression test. Prevalence and confidence intervals were calculated by random-effects meta-analysis, one for each burnout dimension. The heterogeneity of the sample was analysed by Cochran’s Q test and the I^2^ index.

## 3. Results

**Figure 1:**
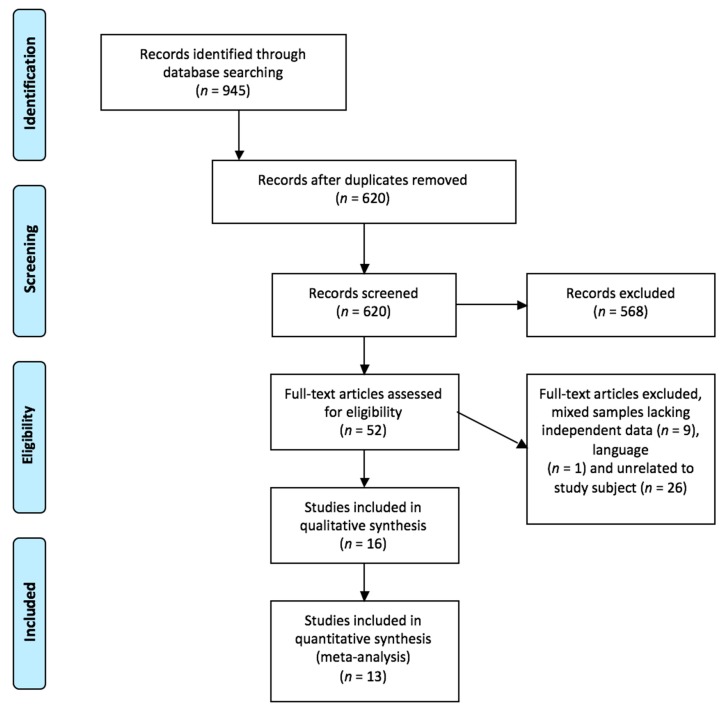
Flow search diagram. Study selection process for the meta-analysis.

In total, 945 relevant studies were located. After reading the titles and abstracts, *n* = 568 records were excluded because they were not related to the study topic, the sample also included women, the study design was not appropriate for this review, or due to the language of publication. After reading the full texts, the final sample was *n* = 16 after eliminating duplicates and applying the inclusion and exclusion criteria. The search and selection process is described in Figure 1. The 37.5% were cohort studies and 50% were carried out in the United States of America. The characteristics of the studies and the HPV risk factors are summarized in Table 1.

### 3.1. Meta-Analysis

**Figure 2:**
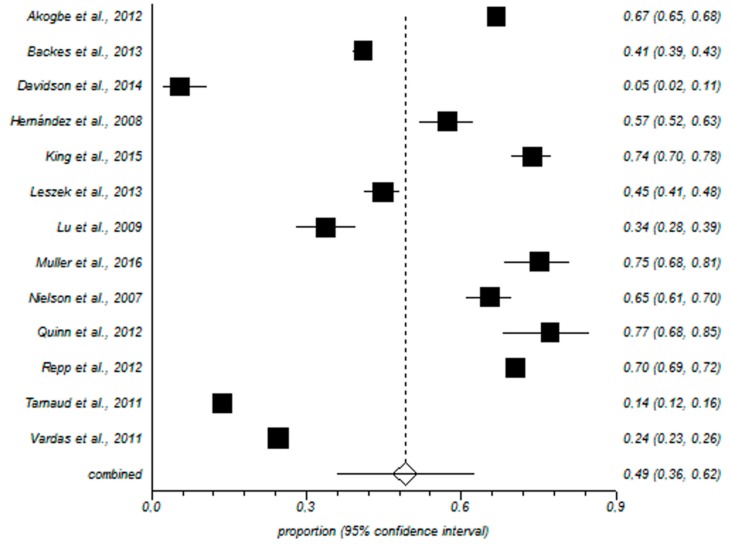
Forest plot of any HPV prevalence. Any HPV Prevalence informed by each study and the meta-analytic estimation.

**Figure 3:**
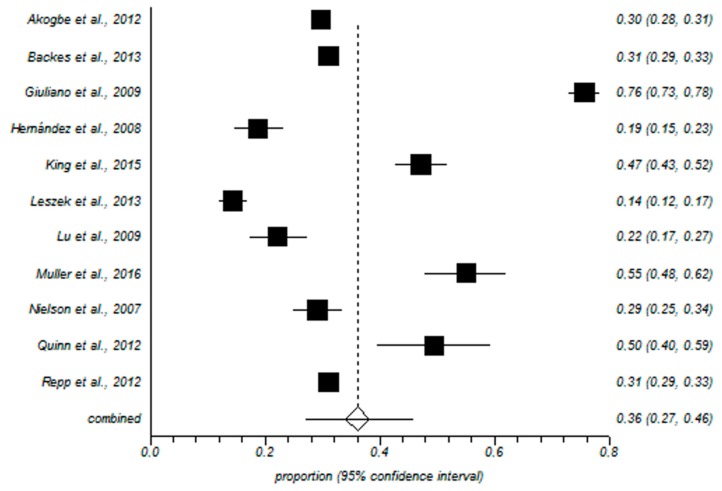
Forest plot of high-risk HPV prevalence. High risk HPV prevalence informed by each study and the meta-analytic estimation.

The meta-analytical estimation of the prevalence of any HPV in males was 49% with a 95% confidence interval of 35–62%. The forest plot is shown in Figure 2. Regarding the prevalence rate of high risk HPV the meta-analytical estimation was 35% with a 95% confidence interval of 26–45%. The forest plot is shown in Figure 3.

In the heterogeneity analyses of the any HPV meta-analysis, the Cochran Q-value was 3555.24 (*p* < 0.001) and the I^2^ index was 99.7%. For the high-risk HPV meta-analysis the Cochran Q-value was 1118.93 (*p* < 0.001) and the I^2^ index 99.1%.

The Egger test of publication bias obtained no statistically significant results, *p* = 0.45 and *p* = 0.57, respectively. We conclude, therefore, that our results are not subject to any publication bias. In the sensitivity analysis, when each of the studies was removed in turn from the sample, the prevalence values did not change in a statistically significant way.

### 3.2. HPV and the Initiation of Sexual Relations or Promiscuity

Several studies have reported a strong association between HPV and promiscuous sex [24,26,27]. According to Giuliano [19], the reduced risk of HPV is significantly associated with older age of sexual initiation; thus, an Odds Ratio (OR) of 0.38 was obtained for any HPV infection among males aged 23–42 compared to those aged ≤13 years. Similarly, Lu et al. [24] reported a lower risk of infection when the age at first sexual intercourse was older (Hazard Ratio 0.9 [95% CI: 0.8–1.0]) and when there was a stable partner (OR 0.55–0.58 for non-oncogenic and oncogenic HPV).

A significantly higher risk of HPV detection was associated with an increased number of lifetime female sexual partners; OR 6.96–9.01 for non-oncogenic HPV (any type) and for oncogenic HPV among men with ≥50 partners versus only one partner [19]. According to Davidson [18], the association between HPV and the number of sexual partners is statistically significant (*p* = 0.027).

Ingles et al. [21] reported that younger men were at higher risk of HPV infection as informed by Lu et al. [24] and Leszek et al. [23].

### 3.3. Circumcision

Various studies have shown that circumcision is a protective factor against HPV infection [16,19,20,24]. Hernandez et al. [20] reported that the prevalence of HPV among circumcised men (50%) was lower than among uncircumcised men (60%). Uncircumcised men had a higher prevalence of HPV infection (46% vs. 29%, adjusted OR, 1.96 (95% CI: 1.02–3.75) and were at higher risk of oncogenic infection (31% vs. 16%, adjusted OR, 2.51 (95% CI: 1.11–5.69) and risk of infection with multiple types of HPV (31% vs. 12%; adjusted OR, 3.56 (95% CI: 1.50–8.50) [26]. 

According to Tarnaud et al. [29], the mechanisms by which circumcised men are less likely to get infected with HPV may be related to a reduction in the acquisition of new infection or to clarification of pre-existing infection, since the absence of foreskin may reduce the risk of auto-reinfection at the urethral site.

Male circumcision is strongly associated with a lower probability of flat penile lesions. Backes et al. [17] reported that circumcised men were much less likely than uncircumcised men to have such lesions (0.7% vs. 26.0%, crude OR = 0.02; 95% CI: 0.003–0.1). These authors concluded that high-risk HPV infection and the high viral load of HPV16/18/31 in the glans were particularly strong risk factors for flat lesions of the penis.

### 3.4. Condom Use

Condom use is considered a protective factor against HPV infection [24,28,30]. According to Nielson et al. [26], consistent condom use is associated with a lower prevalence of HPV. The detection of HPV DNA at anatomical sites that are not covered by the condom may explain the non-reduction of HPV prevalence among condom users in the study by Vardas et al. [30].

### 3.5. Smoking

Several studies have concluded that tobacco use aggravates the risk of HPV infection [24,26]. Current smoking has been associated with a risk of infection by any type of HPV (HR 2.4 [95% CI: 1.3–4.5]) and by non-oncological HPV (HR 2.2 (95% CI: 1.0–4.8]) compared to persons who have never smoked [24].

A stronger association was observed between smoking 10 or more cigarettes per day (compared to smoking 0–9 cigarettes per day) and each of the following outcomes: detection of any HPV (OR 3.0 (95% CI: 1.4–6.4); oncogenic HPV (OR 3.7 (95% CI: 1.6–8.5); and non-oncogenic HPV (OR 2.4 (95% CI: 1.1–5.6]) [26].

### 3.6. Vaccination

According to King et al. [22], based on evidence of current infection, there is a potential benefit in vaccinating men against HPV. The results obtained suggest that expanding the use of the tetravalent vaccine could protect 13–22% men against HPV infection. In addition, it could prevent up to 76% cases of anal cancer. A study conducted in South Africa concluded that targeted interventions and HPV vaccination for children should also be considered, in order to reduce the burden of HPV-related diseases among gay men [25].

### 3.7. Miscellaneous

Education is considered to be a factor related to HPV infection [24]. University or other higher education is significantly associated with a lower risk of acquiring new infection, for any type of HPV (HR 0.3 (95% CI: 0.2–0.7).

According to Ingles et al. [21], genital warts are mainly caused by low-risk types of papillomavirus such as HPV-6 or HPV-11. Quinn et al. [27] and Müller et al. [25] noted that previous studies had shown that anal HPV infection is more common among gay men than among heterosexual men with an anal HPV prevalence being 4–10 times higher among the former. 

Akogbe et al. [15] conducted one of the few multinational studies focusing on racial differences in the prevalence of HPV. In general, the prevalence of any HPV infection (42.3%) and of oncogenic HPV (18.9%) was lowest among Asians and Pacific Islanders. The absolute differences in prevalence between the study population (Asian-American and Asian-Brazilian) and reports from Asian countries may be due to differences in socio-cultural relationships, regarding strict social behaviour, associated with a conservative, restricted sexuality. This behaviour pattern may have been abandoned by Asian-Americans as their culture shifted towards that of the United States.

## 4. Discussion

Due to the association between cervical cancer and HPV, men have not been the focus of HPV research [31]. Notwithstanding, as shown in the results, the HPV prevalence rates in men are not low. Some studies inform that HPV vaccination among men is very low and that most of them think that HPV vaccination is only for females [32]. This, added to the fact that the HPV infection is mostly subclinical, makes men play an important role in the transmission of the infection to the general population acting as reservoirs of the disease [32]. 

Sexual promiscuity, related to the number of sexual partners, in men is an important risk factor due to multiple sexual contacts facilitate contamination by HPV, which is then transmitted to sexual partners [33]. When sexual relations are limited to a small number of partners, this greatly reduces the probability of HPV transmission and therefore the risk of developing cancer. However, the influence of the sexual network depending on its characteristics (such as prevalence of HPV vaccination, condom use, sexual promiscuity and age of the first sexual relationship) should also be taken into account when estimating the risk of HPV. This is especially relevant in women, who are more exposed to HPV infection, which has been suggested to be directly associated with the development of cancer [10].

Some authors argue that the prevalence of HPV infection is associated with age, and hence with the pattern of sexual behaviour in the community. Thus, HPV prevalence is greater at earlier ages (15–25 years) with the initiation of sexual intercourse; later, between 25–40 years of age, there is a marked decrease, after which the prevalence stabilises [34]. However, the percentage of persistence among those aged 25–40 years is higher, which implies an increased risk of pre-neoplastic lesions, due to the above-mentioned trend in this age group [35].

Regarding prevention, penile circumcision appears to be a protective factor against high-risk HPV infection. Circumcised men show a lower risk of HPV infection [36]. In uncircumcised men, the foreskin retracts on the axis during intercourse, and the internal preputial mucous membrane is exposed to vaginal and cervical fluids. In addition, the intact foreskin is vulnerable during intercourse, and may facilitate viral entry, with the subpreputial cavity providing a humid environment that can provide a favourable environment for HPV survival. As a result, circumcision can reduce HPV exposure and access to epidermal basal cells. In addition, the keratinisation of the circumcision scar can reduce the risk of HPV infection [36,37].

Condoms present an impermeable barrier to particles the size of the pathogenic microorganisms of STIs, preventing their transmission and acquisition by avoiding contact between the penis and genital secretions, the mucous membrane and the skin of the sexual partner. Therefore, condoms are considered to be a protective factor against HPV infection [38,39].

The tetravalent HPV vaccine provides effective prevention, as shown by the significant decrease in the rate of immune infection [40]. However, according to researchers, the recent vaccines against high-risk strains of HPV are prohibitively expensive for many of the poorest countries. On the other hand, it has been claimed that the protective effects of circumcision may substitute for HPV vaccines in terms of genotype coverage and target age-group [36].

Smoking is considered to induce added negative effects to HPV infection because cigarette smoke contains chemicals that damage the genetic structure of the human cells. As a result of this damage to the DNA, cells turn into oncogenic cells, which accelerates the appearance of genital lesions and evolution towards cancer [41].

This study has some limitations. First, the number of studies is low because not all the studies did inform about the prevalence of HPV in men or its risk factors without mixing the data with female populations or using other diseases as main subjects of the study. Second, the analysis of the risk factors has been done individually, lacking multivariable analysis. Some variables may influence the other HPV risk factors, e.g., the educational level has been identified by some authors as a predictor of condom use, HPV awareness and HPV vaccination in males [42,43]. Finally, although inclusion criteria were established to find studies with similar populations, a high heterogeneity between studies was found. It may be explained by the diversity of the countries, with different cultural contexts, where the studies have been conducted.

## 5. Conclusions

To our best knowledge, this is the first meta-analysis addressing the prevalence of HPV in the male population and associated risk factors. We have observed a prevalence of 49% in any HPV and of 36% in high risk HPV. Stable relationships, circumcision, condom use and non-smoking are potentially beneficial factors in preventing the development of HPV infection and thus, they should be given serious consideration. The issue of HPV infection in men warrants further research, particularly studies aimed at promoting the provision of a vaccine for this population group and educational campaigns aimed to change sexual behaviour, especially among young men. 

## Figures and Tables

**Table 1:** Characteristic of included studies (*n* = 16).

Author, Year (Country of Study) [Reference]	Sample	Design	HPV Risk Factors	EL	RG
Akogbe et al., 2013 (Brazil, Mexico and USA) [15].	*n* = 3909	Cohort study	Asian/Pacific Islanders had the lowest prevalence of all types of HPV (42.3%) compared to black (66.2%), Mexican (62.3), other (67.3%) and white races (71.5%). Asian/Pacific Islanders had the lowest prevalence of oncogenic HPV (18.9%), compared to black (32.2%), whites (31.4%), Mexican (27.5%) and other (27.1%).	2b	B
Auvert et al., 2009 (South Africa) [16].	*n* = 1264	Clinical trial	Male circumcision protects against high risk-HPV (23.2% prevalence in uncircumcised men against 14% in circumcised men).	2b	B
Backes DM et al., 2012 (Kenya) [17].	*n* = 2509	Clinical trial	Male circumcision was strongly associated with low probability of flat penile lesions (OR = 0.02) and with higher probability of popular lesions (OR = 3.0). Men with flat penile lesions have higher prevalence of the five most common HPV types comparing to men without flat penile lesions.	1a	A
Davidson et al., 2014 (South Africa) [18].	*n* = 128	Cross-sectional study	More sexual partners increase prevalence of oral and oropharyngeal HPV prevalence. No statistically significant differences in HPV prevalence were found regarding age, smoking, alcohol consumption, oral sex, HIV and clinical lesions.	2c	B
Giuliano et al., 2009 (Brazil, México and USA) [19].	*n* = 998	Cohort study	Statistically significant associations (*p* < 0.05): Between 30–34 years, the probability of suffering oncogenic HPV (OR = 1.99) and any type of HPV (OR = 1.81) is higher. Reduced risk of any type of HPV infection in Asians/Pacific Islands (OR = 0.18) and in mixed races (OR = 0.74). Reduced risk of oncogenic HPV in Asians/Pacific Islands (OR = 0.32). Increased risk of oncogenic HPV (OR = 2.18) and of any type of HPV (OR = 2.12) in Brazil. Higher risk of oncogenic HPV (OR = 2.27) and of any type of HPV (OR = 2.63) in divorced/separated. Lower risk of oncogenic HPV (OR = 0.70) in non-smokers. Increased risk of oncogenic HPV (OR = 9.01) and of any type of HPV (OR = 8.15) is related to a greater number of couples. Lower risk of oncogenic HPV (OR = 0.30) and of any type of HPV (OR = 0.44) if they have not had sex in the last 3 months and Increased risk of oncogenic HPV (OR = 3.43) and of any type of HPV (OR = 3.05) is related to a greater number of couples in the last 3 months. Greater risk of oncogenic HPV (OR = 1.45) and of any type of HPV (OR = 1.40) if they have had anal sex. Lower risk of oncogenic HPV (OR = 0.47) and of any type of HPV (OR = 0.52) if they have never been diagnosed with sexually transmitted disease. Increased risk of any type of HPV (OR = 1.57) if they have genital herpes. Increased risk of oncogenic HPV (OR = 2.27) and for any type of HPV (OR = 2.19) if they have had partners with genital warts.	2b	B
Hernández et al., 2008 (USA) [20].	*n* = 351	Cohort study	Smoking, sex with men, lifetime number of female sex partners, history of genital warts and circumcision are associated with HPV. History of sex with men was inversely associated with HPV infection of the shaft (OR = 0.44). Lifetime number of female sex partners was positively associated with HPV infection of the shaft and scrotum (OR = 6.93). Uncircumcised men have higher prevalence rate of HPV of the glans (OR = 1.96) and oncogenic infection (OR = 2.51).	2b	B
Ingles et al., 2015 (USA, Brazil, México) [21].	*n* = 2754	Cohort study	High-risk type HPV is more prevalent in penile intraepithelial neoplasia (85.7%) and other genital external lesions (21%) than in condiloma (8.2%).	2b	B
King et al., 2015 (England) [22].	*n* = 511	Cross-sectional study	Higher HPV prevalence rate (92.6%) in HIV-positive men who have sex with men compare with HIV-negative men who have sex with men (71.1%). There is a 4.7% increase in the odd of HPV infection per year when modeling age as a continuous variable (18–40 years). When treating age as a categorical variable there are no significant differences in HPV infection.	2c	B
Leszek et al., 2013 (Poland) [23].	*n* = 820	Cross-sectional study	Higher risk of HVP infection with more than three sexual partners in the last 12 months (OR = 1.44). HPV infection is greater in men younger than 34 years (OR = 1.08). Higher HPV infection in men who do not use condoms (OR = 1.86).	2c	B
Lu et al., 2009 (USA) [24].	*n* = 377	Cohort study	There is a reduced risk of HPV infection with having a college education or higher (Hazard rate = 0.3) and with older age at first sexual intercourse (Hazard rate = 0.9). There is an increased risk of HPV infection in smokers (Hazard rate = 2.4).	2b	B
Müller et al., 2016 (South Africa) [25].	*n* = 200	Cross-sectional study	Men with HIV have higher prevalence rate of anal and oropharyngeal HPV. Men who have sex with men are more likely to have anal HPV than men who have sex with woman (85% vs. 26.8%). HPV infection is significantly associated with receiving an income, having sex with men only, engaging in group sex in their lifetime, being HIV positive and practicing receptive anal sex.	2c	B
Nielson et al., 2007 (USA) [26].	*n* = 463	Cross-sectional study	Sociodemographic factors are not related to HPV infection. Smoking is associated with HPV (OR = 1.8). Smoking 10 or more cigarettes per day is associated with HPV (OR = 3.0). Using condoms at least half time reduce HPV (OR = 0.5). Sexual factors such as high number of lifetime female sex partners, female partners on the past 3 months, and an increased frequency of intercourse in the past month and 3 months are significantly related to HPV. Current presence of genital warts (OR = 4.5) and having a female parte with abnormal pap smear (OR = 2.2) are related with HPV.	2c	B
Quinn et al., 2012 (Peru) [27].	*n* = 105	Cross-sectional study	Being primary receptive as sex role is related with a higher prevalence of HPV, 56%, vs. 4% in insertive. Having exclusively men as sexual partners is related with higher HPV prevalence (92%) vs. 17.2% when having men and women. High risk HPV is related to not having a stable relationship, have sex work as primary income and having a greater number of life partners.	2c	B
Repp et al., 2012 (Brazil, Mexico, USA) [28].	*n* = 2621	Cross-sectional study	There are statistically different prevalence rates of any HPV for men that always use condom (65.6%) and men do not always use it (71.9%). There are statistically different prevalence rates of any oncogenic HPV for men that always use condom (29.6%) and men do not always use it (35.4%).	2c	B
Tarnaud et al., 2011 (South Africa) [29].	*n* = 3274	Cohort study	Low risk HPV genotypes significantly increase with the number of lifetime sexual partners. HIV and Herpes Virus are associated with an increase in low risk HPV genotypes. Number of low risk HPV genotypes decrease in participants with primary education and those that use condom.	2b	B
Vardas et al., 2011 (5 continents) [30].	*n* = 3463	Cross-sectional study	Less condom use is associated with an increased risk of HPV (OR = 1.7). More than 3 lifetime female sexual partners increase prevalence HPV DNA detection (OR = 4.5). Africa has the highest risk of HPV (OR = 3.7).	1a	A

*Note*: EL = Evidence level; HPV = Human Papillomavirus; OR = Odd ratio; RG = Recommendation grade; USA = United States of America.
